# Natural Parasite Infection Affects the Tolerance but Not the Response to a Simulated Secondary Parasite Infection

**DOI:** 10.1371/journal.pone.0052077

**Published:** 2012-12-27

**Authors:** Heike Lutermann, Chimoné Bodenstein, Nigel C. Bennett

**Affiliations:** Department of Zoology and Entomology, University of Pretoria, Private Bag X20, Hatfield, South Africa; University of Plymouth, United Kingdom

## Abstract

Parasites deplete the resources of their host and can consequently affect the investment in competing traits (e.g. reproduction and immune defence). The immunocompetence handicap hypothesis posits that testosterone (T) mediates trade-offs between parasite defence and reproductive investment by suppressing immune function in male vertebrates while more recently a role for glucocorticoids (e.g. cortisol (C)) in resource allocation has been suggested. These hypotheses however, have not always found support in wild animals, possibly because most studies focus on a single parasite species, whereas infections with multiple parasites are the rule in nature. We measured body mass, T- and C-levels of wild male highveld mole-rats (*Cryptomys hottentotus pretoriae*) naturally uninfected or infected with a cestode (*Mathevotaenia* sp.) right after capture. Subsequently, we injected animals subcutaneously with a lipopolysaccharide (LPS) to simulate a bacterial infection and recorded changes in body mass, food intake, haematological parameters and hormone levels. As a control, animals were injected with saline. Natural infection neither affected initial body mass nor C-levels, whereas infected males had significantly reduced T-levels. We observed significant reductions in food intake, body mass and T in response to LPS but not saline while C increased. However, this response did not vary with infection status. In contrast, final body mass and some haematological parameters were significantly lowered in infected males. Our results suggest that naturally infected males are able to compensate for resource depletion by physiological adjustments. However, this leaves them less tolerant to the challenges of a secondary infection.

## Introduction

Life-history theory proposes that the resources available to an organism are limited and consequently, trade-offs between the investment in different life-history traits such as maintenance, reproduction and immune defence can be expected [1]. Parasites can greatly affect the amount of resources (e.g. energy) available to their host either through direct competition, physiological costs caused by the immune responses they induce or costly behavioural responses such as grooming [2]–[5]. In accordance with a resource depletion by parasites, body mass loss and reductions in fat stores have been reported for infected individuals compared to their uninfected conspecifics in a number of vertebrate species [2], [6], [7]. However, hosts may counter such detrimental effects of parasites with morphological and physiological adjustments such as increases in resorptive surface and/or changes in organ size and consequently body mass loss or fat depletion have not always been observed [8]–[10].

The immunocompetence handicap hypothesis (ICHH) posits that in male vertebrates the trade-off between parasite infection and reproductive investment may be mediated by the hormone testosterone [11]. It proposes that testosterone (T), required for the expression of sexual traits, suppresses the immune system resulting in a lower ability to combat pathogen and parasite infections. In accordance with this hypothesis, a reduction of T-levels in response to parasite infection has been reported in a number of studies that can result in a lowered propensity to engage in sexual behaviours and decreased fertility [12]–[14]. However, this is not always the case and the direction of such correlations may vary greatly, particularly if more multiple parasite species are considered [15]–[17]. Hence, it has been proposed that the trade-off between the expression of sexual traits and parasite defence may be mediated by glucocorticoids (GCs) such as cortisol and corticosterone instead [18]–[20]. Indeed, GCs can have both, immune-enhancing and suppressing properties [21]–[24]. In support of the former, negative correlations between GC-levels and parasite burdens observed in a number of studies have frequently been interpreted as an indicator of immunosuppressive properties of GCs [25]–[27]. However, the release of GCs mobilizes resources in times of need and hence, elevated GC-levels during parasite infection may alternatively indicate the mobilization of resources for parasite defence [23], [24].

While a vast number of studies investigating the relationship between steroid hormones and parasite burdens consider only a single parasite species, infections with more than one parasite (i.e. coinfection or multiple parasites) are the rule rather than the exception in nature [28], [29]. Hence, observed patterns of androgen hormones and resource distribution in wild organisms are likely to be a result of the multiple parasite challenges a host faces under natural conditions and at different points in time. This may also account for the mixed results with regard to relationships between individual parasite species, androgens and body condition reported in some studies [16], [17]. Parasites are entirely dependent on their hosts to provide their trophic needs. As a result, two parasites populating the same host may directly compete for host resources and the outcome of this competition can be determined by their competitive abilities without this being reflected in the hosts’ physiology [30]. However, different parasites do not necessarily exploit the same host niche (e.g. body compartment) but can still affect each other by the immune responses they trigger in their hosts. Such interactions have been extensively studied in laboratory models [31]–[33] and suggest the mutual inhibition of different types of T-helper (Th) cells that target either micro- (Th1) or macroparasites (Th2) [34]–[36]. Similar studies in wild organisms are urgently required [37] and one of the few already conducted suggest that the activation of the Th2 response by helminth can result in a downregulation of the Th1 response that in turn increases the probability of a successful microparasite infection in the wild [38]. However, the predominance of studies in laboratory rodents stresses the need for similar studies in wild organisms. In addition, laboratory studies are usually limited to immunological aspects and do not consider steroid hormone as mediators.

The current study aimed to evaluate the effects of a chronic infection on body mass, T, cortisol (C) and immune parameters and how such an infection modulates sickness behaviours triggered by a simulated secondary infection in the highveld mole-rat (*Cryptomys hottentotus pretoriae*). In this group-living, subterranean rodent, reproduction is limited to a small number of males per group and births occur mostly from July to November. Regardless, all males are fully reproductively competent and retain their reproductive function throughout the year [39], [40]. We firstly, assessed body mass, T and C-levels for males naturally uninfected or infected by *Mathevotaenia* sp. (i.e. chronic infection), the most prevalent parasite of the study species [41]. Secondly, we then simulated a secondary bacterial infection by subcutaneous injection of lipopolysaccharide (LPS). LPS is a cell-wall component of gram-negative bacteria causing activation of the immune system and sickness behaviours (e.g. anorexia, lethargy) without replicating in the host organism [42]. This allows measuring the costs of the immune response independent of additional pathogenic effects an infectious organism may have on its host. In addition to recording changes in food intake, we repeated the body mass and hormonal measurements and assessed immune function by counting blood parameters and measured organ masses. We hypothesized (i) that males with a chronic infection would have a lower body mass and T-levels but elevated C-levels compared to uninfected males. Furthermore, (ii) we predicted reductions in food intake, body mass and T and concomitant increases in C in response to LPS-administration. However, we expected these effects to be weaker for males with a chronic parasite infection due to indirect competition between parasites. Lastly, (iii) we anticipated evidence for a greater investment in immune function or parasite associated stress for naturally infected males in the haematological parameters (e.g reductions in red blood cells and lymphocytes, increases in neutrophils [43]) and possibly immune organ size (i.e. spleen) while fat deposits should be reduced compared to uninfected males.

## Results

### Effects of Chronic Helminth Infection on Body Mass

**Figure 1:**
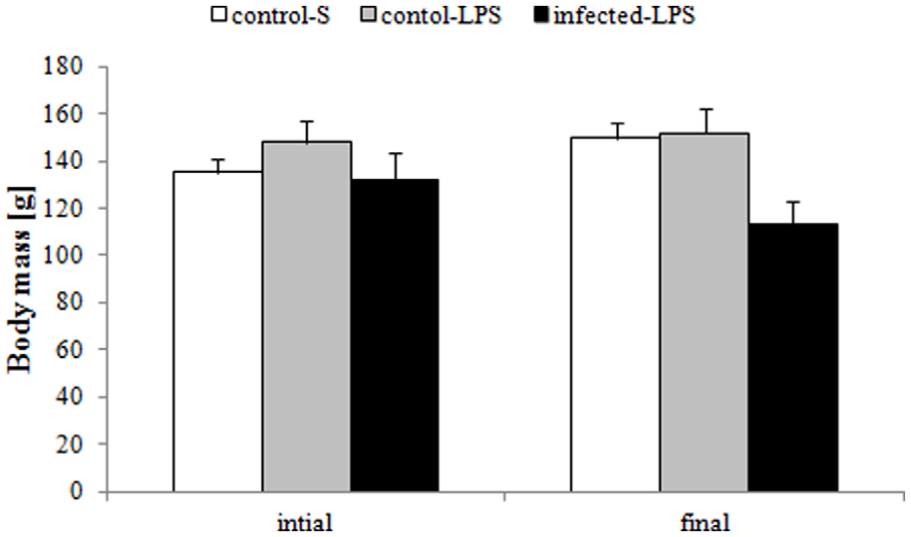
Comparison of initial and final body mass between control-S (open bars, n = 8), control-LPS (light grey bars, n = 12) and infected-LPS (dark grey bars, n = 9) individuals. Displayed are means ± SE.

The GLMM showed that neither treatment (F_1,25_ = 0.21, p = 0.6516) nor time of measurement (F_1,25_ = 0.00, P = 0.9590) significantly affected body mass (Figure 1). In contrast, infected males were significantly lighter than uninfected ones (F_1,25_ = 6.03, p = 0.0213). The interaction between treatment and time was significant (F_1,25_ = 7.62, p = 0.0107) with males receiving saline exhibiting no significant changes in body mass from initial to final measurement (LSD: p = 0.510) while LPS-treated males tended to lose body mass (LSD: p = 0.073). However, there were no significant differences in body mass between the treatments for either initial (LSD: p = 0.217) or final body mass (LSD: p = 0.828). The interaction between parasite and body mass was significant (F_1,25_ = 9.41, p = 0.0051) and while uninfected males significantly gained body mass between the first and the final measurement (LSD: p<0.0001), infected males lost body mass during the same period (LSD: p = 0.053). Consequently, body mass did not differ significantly with parasite status during the initial measurement (LSD: p = 0.269) while uninfected males were significantly heavier than infected males during the final measurement (LSD: p = 0.003). The % body mass loss differed significantly between groups (χ^2^ = 14.87, df = 2, p = 0.001) and was significantly greater for infected-LPS (−13.3±6.4%) compared to control-S (11.1±4.6%, LSD: p<0.0001) and control-LPS animals (3.0±4.1%, LSD: p = 0.006) while it did not differ significantly between control-S and control-LPS animals (LSD: p = 0.169).

### Food Intake and Morphological Responses to Simulated Infection

**Figure 2:**
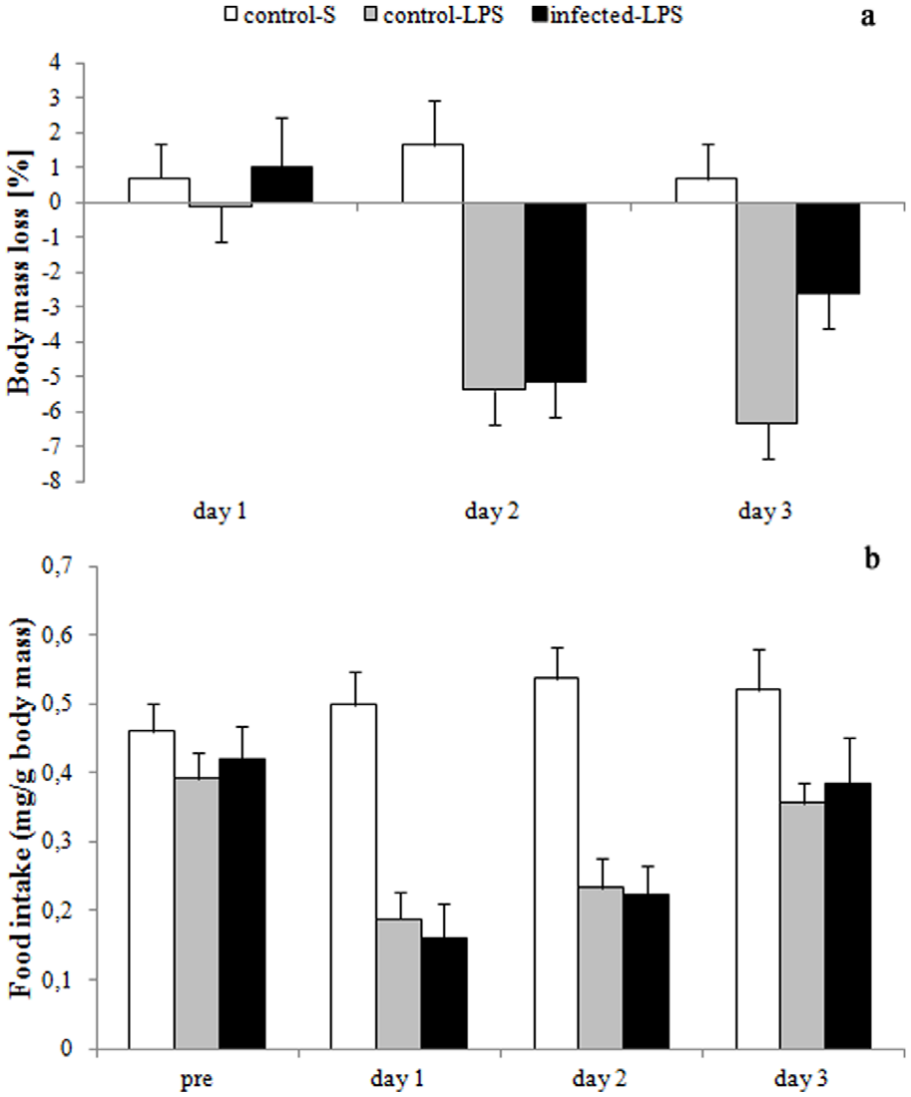
Effects of LPS on a) body mass loss and b) food intake on day 1, 2 and 3 post-treatment compared to the control period (pre) for control-S (open bars, n = 8), control-LPS (light grey bars, n = 12) and infected-LPS (dark grey bars, n = 9) males. Displayed are means ± SE.

The loss of body mass in response to the administration of saline or LPS differed significantly between days (F_2,50_ = 14.30, p<0.0001, Figure 2a) with body mass loss increasing significantly from day 1 to day 2 post-treatment (LSD: p = 0.003) but not between day 2 and 3 post-injection (LSD: p = 0.975). Body mass loss was significantly greater for animals injected with LPS compared to those injected with saline (F_1,25_ = 10.32, p = 0.0036, Figure 2a). However, it was not significantly affected by parasite status (F_1,25_ = 1.35, p = 0.257). The interaction between day and treatment was significant (F_2,50_ = 7.51, p = 0.0014, Figure 2a). For animals injected with saline, body mass loss did not differ significantly between days (p≥0.44). In contrast, for animals challenged with LPS body mass loss increased significantly between day 1 and 2 post-injection (LSD: p<0.0001) but not between day 2 and 3 (LSD: p = 0.493). Consequently, body mass loss did not differ between treatments for day 1 (LSD: p = 0.516) but it was significantly greater for animals receiving LPS than saline on day 2 (LSD: p<0.0001) and day 3 (LSD: p<0.0001). The interaction between parasite status and day did not significantly affect body mass loss (F_2,50_ = 1.72, P = 0.1888). The power analysis confirms that the observed lack of parasite effects is not compromised by the sample size (1–β = 0.986).

The rate of food intake was significantly affected by experimental day (F_3,72_ = 12.77, p<0.0001, Figure 2b). It dropped significantly from the pre-treatment average to day 1 post-injection (LSD: p = 0.002) and increased again significantly from day 2 to day 3 post-treatment (LSD: p = 0.045). However, there was no significant difference in food intake between day 1 and day 2 post-injection (LSD: p = 0.345). Food intake was significantly greater for individuals injected with saline compared to those treated with LPS (F_1,25_ = 21.80, p = 0.0001) but was not affected by parasite status (F_1,25_ = 0.03, p = 0.8681). The interaction between experimental day and treatment was significant (F_3,72_ = 8.67, p = 0.0001). Food intake did not vary significantly between days for animals receiving a saline injection (p≥0.50). In contrast, after LPS treatment individuals significantly reduced their food intake from the pre-treatment mean to day 1 (LSD: p<0.0001) and significantly increased it again from day 2 to 3 (LSD: p = 0.002). It did not change significantly from day 1 to day 2 (LSD: p = 0.095). Treatment had no significant effect on food intake prior to injection (LSD: p = 0.190) but food intake was consistently greater during all days post-injection for saline treated animals (p≤0.009). The interaction between parasite status and day was not significant (F_3,72_ = 0.30, p = 0.8232). However, the latter results should be treated with caution as the power analysis suggested low statistical (1–β = 0.077).

### Effects of Infection and Treatment on Hormone Levels

**Figure 3:**
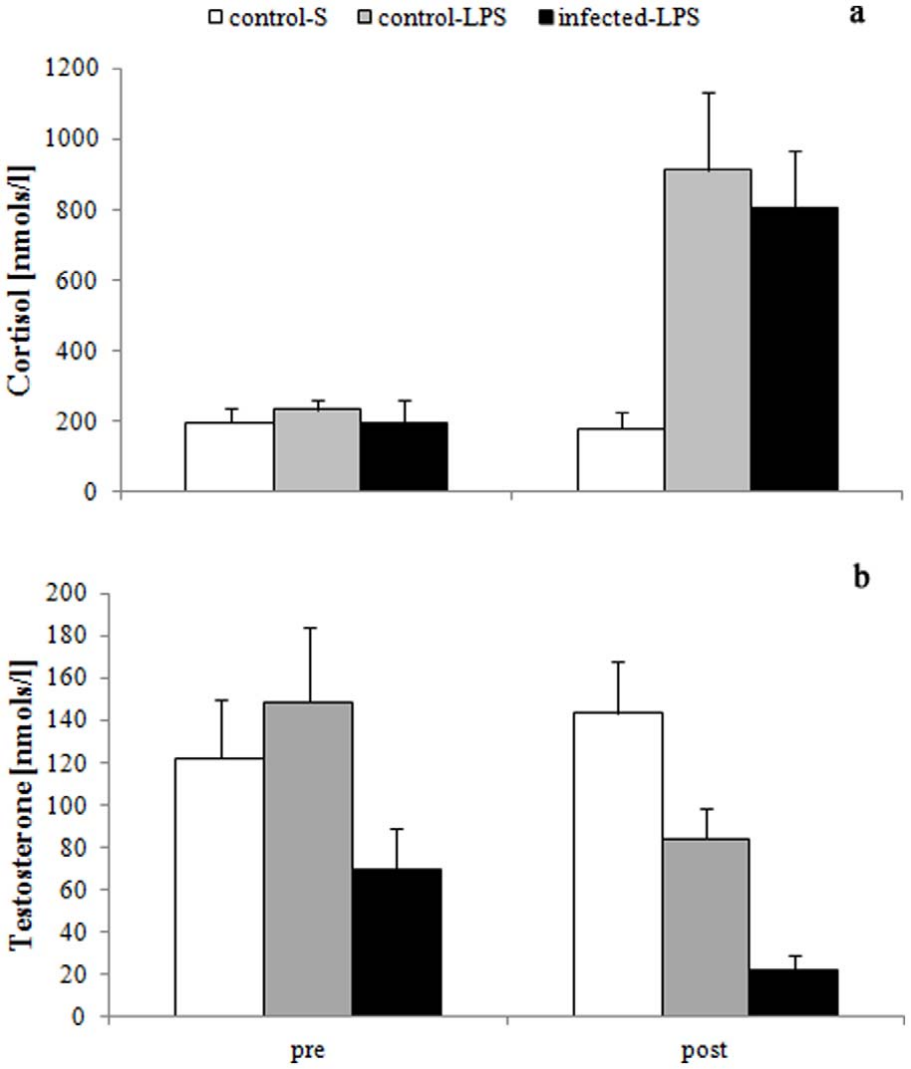
Mean a) cortisol and b) testosterone levels in control-S (open bars, n = 8), control-LPS (light grey bars, n = 12) and infected-LPS (dark grey bars, n = 9) before (pre) and after (post) injection of LPS. Displayed are means ± SE.

Cortisol levels did not differ significantly between treatments (F_1,27_ = 3.52, p = 0.0713) or with parasite status (F_1,27_ = 0.02, p = 0.9006). In contrast, C was significantly elevated after the injection compared to prior to treatment (F_1,23_ = 31.15, p<0.0001, Figure 3a). The interaction between treatment and time was significant (F_1,23_ = 14.12, p = 0.0010) with C-levels increasing significantly in response to injection for LPS-treated (LSD: p<0.0001) but not saline-treated males (LSD: p = 0.600). While the initial C-levels did not differ significantly between treatments (LSD: 0.259) they were significantly lower for saline vs. LPS-injected individuals after the challenge (LSD: p = 0.001). The interaction between parasite status and time was not significant (F_1,23_ = 0.19, p = 0.6681). Power analysis confirmed that the lack of an effect of parasite status was not simply an artefact of sample size limitations (1–β = 0.999).

Testosterone levels were significantly lower in males receiving LPS compared to those receiving saline (F_1,27_ = 4.51, p = 0.0430). Furthermore, they were significantly greater for uninfected compared to infected males (F_1,27_ = 7.71, p = 0.0099, Figure 3b) while they did not vary significantly with time (F_1,23_ = 3.33, p = 0.0811). Although T-levels were reduced in response to LPS-administration but not saline (Figure 3b), the interaction term between treatment and time failed to reach significance (F_1,23_ = 3.70, p = 0.0669). The interaction between parasite status and time was not significant (F_1,17_ = 0.12, p = 0.7339).

### Effects of a Helminth Infection and Treatment on Organ Mass

When comparing absolute mass, the livers, kidneys and spleens of infected males (liver: 5.3±1.2 g, kidney: 1.0±0.0 g, lg spleen: −1.0±0.1 g) were significantly lighter than those of uninfected males (liver: 8.2±0.7 g, χ^2^ = 5.25, p = 0.022; kidney: 1.1±0.1 g, χ^2^ = 3.79, p = 0.051; lg spleen: −0.7±0.1 g, χ^2^ = 3.92, p = 0.048). In addition, kidney mass was significantly heavier for saline injected males (1.1±0.1 g) compared to infected animals (1.0±0.1 g, χ^2^ = 4.36, p = 0.037) while it affected none of the other organ masses (p≥0.50). Neither heart nor fat mass were significantly affected by either parasite status (p≥0.082) or treatment (p≥0.084). However, all organ masses increased significantly with body mass (p≤0.006) and when controlling for body mass, none of these organ masses differed significantly between uninfected and infected males (p≥0.63). Similarly, with the exception of kidney mass (χ^2^ = 11.21, p = 0.001), all organ masses were not significantly affected by treatment (p≥0.51).

### Parasite and Treatment Effects on Immunological Parameters

In accordance with our hypothesis, animals harbouring cestodes exhibited significantly reduced RBCs (χ^2^ = 3.97, df = 1, p = 0.046), haematocrits (χ^2^ = 5.68, df = 1, p = 0.017), lymphocyte (χ^2^ = 9.45, df = 1, p = 0.002) and monocyte counts (χ^2^ = 4.28, df = 1, p = 0.039) compared to uninfected males (Table 1). In contrast, the proportion of neutrophils was significantly elevated in infected compared to uninfected individuals (χ^2^ = 10.08, df = 1, p = 0.001) as predicted. Accordingly, the N:L values were significantly greater in infected compared to uninfected animals (χ^2^ = 10.96, df = 1, p = 0.001, Table 1). Neither the proportion of eosinophils or basophils differed significantly between animals of different parasite status (p≥0.31). While the proportion of monocytes was significantly elevated after LPS-administration compared to saline controls (χ^2^ = 6.34, df = 1, p = 0.012) such a treatment effect was not observed for any of the other parameters (p≥0.15).

**Table 1:** Haematological parameters for control-S, control-LPS and infected-LPS male highveld mole-rats.

Parameter	Control S (n = 8)	Control LPS (n = 11)	Infested LPS (n = 8)
RBC (x 10e12/l)*	8.0±0.4	8.4±0.2	7.6±0.3
Haematocrit (l/l)*	0.49±0.01	0.51±0.01	0.46±0.02
Neutrophils total (%)*	60.0±2.1	60.4±3.5	75.0±3.1
Lymphocytes (%)*	37.0±2.7	33.1±3.1	21.1±3.0
N:L*	1.7±0.2	2.1±0.4	4.3±0.8
Monocytes (%)*	1.8±0.6	4.8±1.1	2.9±0.8
Eosinophils (%)	0.6±0.4	0.9±0.3	0.5±0.3
Basophils (%)	0.8±0.5	0.8±0.4	0.5±0.3

* : parameter differs significantly between groups.

## Discussion

Despite the costs that are by definition associated with parasites and pathogens, few of the parameters measured in the current study appear to indicate such costs. However, detrimental effects of the chronic infection were apparent in T-levels and haematological parameters. Moreover, although initial body mass did not appear to indicate detrimental effects of the helminth infection, the simulated bacterial infection resulted in a significantly reduced final body mass suggesting a reduced ability of infected individuals to deal with a secondary infection. These results indicate that animals may be able to compensate for the costs imposed by parasites to a certain extent and that an assessment of such costs requires both, short and long-term measurements of a multitude of variables in wild organisms.

### Effects of a Helminth Infection on Body and Organ Mass

Contrary to our hypothesis, initial body mass did not differ significantly with parasite status among male highveld mole-rats in our study (Figure 1). This contrasts with a previous study in this species that reports an association with greater body mass and the *Mathevotaenia* spec. [41]. However, since the current study only considered the larger sex (i.e. males) while [41] sampled entire colonies this may be attributable to the study design. Alternatively, the lack of parasite status effects on initial body mass could suggest that *Mathevotaenia* sp. is a relatively benign parasite that causes little costs to its host. Conversely, the overall body mass loss of infected males despite *ad libitum* food availability strongly suggests that the costs associated with this cestode infection can be substantial and may affect the ability to cope with novel infections in the wild. That the long-term body mass loss is indeed related to the cestode infection is corroborated by the ability of control-LPS males to maintain their body mass similar to what can be observed in control-S males. However, phenotypic plasticity could enable an infected individual to compensate for the costs inflicted by the parasite [8]–[10], [44]. Increases in food intake could make up for additional energy requirements resulting from parasite infection [2], [3], [5]. Our food intake reference data, however, does not support this hypothesis as food intake prior to LPS-administration was not significantly affected by infection status. Conversely, individuals infected by helminths may not be required to increase their food intake, if they adjust the efficiency of their gastrointestinal (GI) tract, by increasing the resorptive surface and/or resorption rates, to compensate for food depletion and tissue damage caused by helminths [8], [9], [44]. Since we have not measured these parameters in our study, we cannot exclude this possibility. However, our data does not provide evidence for an enlargement of organs other than the GI tract (e.g. spleen or liver) that has repeatedly been reported in response to helminth infection [8], [10], [44]. Nonetheless, changes in organ sizes is affected by the duration of an infection and more apparent early during an infection while it may be attenuated during chronic infection [8] such as the cestode infection in our study animals. At the same time, the kidneys were the only organs suggesting a treatment effect. This may be related to the central role of renal tissues in cytokine and GC regulation [45], [46].

### Physiological Responses to a Natural Helminth Infection

Our hypothesis that C-levels would be increased in infected-LPS highveld mole-rat males was not supported (Figure 3a). Similar to the lack in body mass differences the chronic nature of the cestode infection could account for this observation. While highveld mole-rats clearly responded with increases in C to an acute infection (i.e. LPS-challenge), the negative effects of sustained elevated C-levels have frequently been stressed [47], [48]. Accordingly, it has been suggested that corticosteroids will be elevated in response to an acute environmental stressor to initiate facultative physiological and behavioural adjustments in an individual [49]. Once allostasis (physiological balance) is achieved by shunting resources from non-essential function (e.g. reproduction) corticosteroid levels should decrease again [48]. Cortisol has an antagonistic effect on the acute phase responses [21], [46] and consequently, the similar C-levels between males of all three experimental groups prior to injection would also explain why the predicted attenuated response to the LPS-challenge was not observed in infected males. The significant decrease in initial T-levels recorded in infected males compared to both groups of infected males could indicate a resource trade-off and is suggestive of a decreased investment in reproductive traits for infected males [11]. Although this may not directly affect a males’ survival the frequently observed correlation between T-level, testes size and reproductive success would still suggest fitness impairment for infected males [50].

At the conclusion of the experimental period, we found a reduction in RBCs, haematocrit levels and lymphocytes in infected males, while their neutrophil counts and consequently the N:L were increased compared to uninfected males. These are characteristics indicative of deficient nutrition and parasite infection and an immunological response that may be triggered by tissue damage caused by gastrointestinal helminths [43], [51], [52]. This is further corroborated by the lack of treatment effects on any of these parameters and suggests a functional link between the chronic infection and these immunological parameters. In addition, only monocytes were affected by treatment and their elevation is a typical indicator of inflammation due to their role in cytokine regulation [45]. The concomitant differences in T-levels between infected and uninfected males furthermore suggests that the reduction in T-levels permitted the former to maintain their body mass despite their investment in immune function as suggested by the ICHH [11].

### Food Intake and Morphological Responses to Simulated Infection

The injection of LPS resulted in anorexia and the significant reduction in body mass post-injection that is typical for the acute phase response (APR, Figure 2) [42], [45]. Although an inhibition of the Th1 and hence a lower APR were expected as a result of the cestode induced activation of the Th2 system, the APR did not differ significantly between control-LPS and infected-LPS but was not observed in control-S males. The power analysis suggested that the food intake data may have been compromised by sample size limitations. However, this did not apply to body mass loss and hence it appears unlikely that the comparable response to LPS-administration between control-LPS and infected-LPS males is solely attributable to a small sample size. Hence our observation may indicate that the APR is highly stereotyped and not affected by energetic constraints. However, a number of studies clearly contradict this hypothesis and show that reductions in fat reserves result in attenuated APR responses [53]–[56]. In addition, another component of the APR (i.e. fever) varies markedly with sex and breeding status in highveld mole-rats [57] suggesting that a similar modulation can be expected for other characteristics of the APR. Nonetheless, if the extent of energy resources available determines the expression of anorexia and body mass loss during the APR, the comparable energy resources (i.e. fat mass) observed for uninfected and infected males could account for the similar responses in both groups.

### Physiological Responses to Simulated Infection

Similar to the behavioural and body mass response to treatment, the hormonal changes did not differ with infection status of a male. However, they were observed in response to LPS-injection but not saline. Consequently they were clearly triggered by the simulated infection. As predicted C-levels increased and T-levels decreased after LPS-treatment lending support to the hypothesis that both C and T are involved in mediating trade-offs between investment in immune function and reproduction [18]–[20]. The activation of the immune system is energetically costly and C is considered a hormone responsible for the mobilisation of energy resources [23]. In addition, it is known to act as an important negative feedback mechanism of the APR that is not affected by body mass loss [54], [58]. Accordingly, the similar C-response in males may indicate a general mechanism.

As predicted, males exhibited significant reductions of T-levels after LPS but not saline injection, corroborating the hypothesis that males trade-off investment in reproduction (i.e. T-levels) and immune function and similar correlations have been reported for other vertebrate species [11], [13], [14]. The increment at which control-LPS and infected-LPS males reduced their T-levels was comparable and this may suggest that a fixed amount of redistribution is necessary to mobilize the energy required for an APR. Interestingly, the post-treatment T-levels for control-LPS males were similar to the pre-treatment levels of infected-LPS males suggesting that the costs of a chronic helminth infection may be equivalent to those of a simulated acute bacterial infection. Post-treatment T-levels were significantly negatively correlated with the N:L (R_S_ = -0.522, n = 18, p = 0.026) implying that there is indeed an antagonistic relationship between this steroid hormone and investment into the immune system.

### Conclusion

In the current study we aimed to assess the costs of a natural parasite infection (chronic) as well as the costs of the immune response triggered by a simulated bacterial infection (acute) and possible trade-offs between the two by measuring morphological and physiological parameters as well as food intake in male highveld mole-rats. Our results suggest that males may be able to compensate for the effects of a chronic helminth infection by trading off their investment in reproductive traits (i.e. T-levels) with that in immune function. Furthermore, our results indicate that a chronic infection does not necessarily impair the immune response to a secondary infection. However, we found evidence that infected animals are less able to cope with the additional challenge posed by a secondary infection on a medium-term basis and this could have significant fitness implications in the wild.

## Materials and Methods

### Ethics Statement

All procedures were approved by the Ethics Committee of the University of Pretoria (EC001-10).

### Capture and Housing

Highveld mole-rats were captured from the Tshwane area (S25°46′35.45″ E28°21′37.34″), South Africa between February and September 2010 using Hickman live-traps baited with sweet potato. Traps were checked three times daily, and the bait was replaced on a daily basis. Captured animals were initially housed together in their colonies in plastic crates (49.5 x 28 cm) and were provided with wood shavings and paper towels as bedding. Animals were maintained on an *ad libitum* diet of fresh sweet potato in a climate controlled room at 25±1°C and with a light cycle of 12∶12 h LD. Within a week of capture animals were weighed (initial body mass) to the nearest 0.1 mg (Scout Pro SPU123, Ohaus Corporation, USA) and transferred to individual crates. Only adult males were used in the current study.

### Parasite Assessment

Droppings deposited on the wire mesh base during urine collection were examined for the presence of proglottids of *Mathevotaenia* sp. for the classification of infected and uninfected males. Due to the large size of the proglottids of Mathevotaenia sp. such a visual inspection allows a reliable identification of this cestode and this method has been shown to have a high accuracy [41]. To further ensure that no male classified as uninfected did harbour any cestodes, we treated these males with 0.1 ml/kg ivermectin dissolved in saline solution and injected subcutaneously. Infected males received an injection with the equivalent amount of saline solution. In addition, all males were treated against ectoparasites by applying flea powder. In accordance with the manufacturer’s instructions, we allowed two weeks to elapse before any further experimental procedures were conducted. The effectiveness of these procedures was confirmed by post-mortem examination of the gastrointestinal tract and parasite identification was confirmed by the Onderstepoort Veterinary Institute, Pretoria, South Africa. A total of 29 males (20 uninfected, 9 infected) were collected for this study. No cestodes were found in any of the males classified as uninfected while infected individuals harboured between one and 36 cestodes (Median: 9, intensity: 12.8±4.4). Although these were naturally acquired cestodes and we have consequently no means of determining the actual duration of this infection the fully developed cestodes suggest that animals were chronically infected over a period of weeks to month [59].

### Urine Collection and Hormone Assays

For hormonal analyses we collected urine from males by placing them in a plastic cylinder (height: 20 cm; radius: 9.6 cm) with a wire mesh base and a collection tray underneath the cylinder. Each volume of urine deposited was collected with a pipette, transferred to an Eppendorf tube and frozen at −20°C until assayed. Urine collection was conducted before saline/LPS-administration and repeated once the experimental part of this study was concluded (see below). The urinary T and C concentrations were measured using Coat-a-Count kits (Diagnostic Products Corporation, Los Angeles, California) for T and C, respectively. The antiserum of the former is highly specific for T and has a low cross-reactivity with other naturally occurring steroids, except dihydrotestosterone, which is <5%. The assays were validated by testing for parallelism using doubling dilutions of mole-rat urine over a dilution range (1∶1 to 1∶64). The slope of the line was then compared using an ANCOVA test. The sensitivity of the assay was 2.2 nmols/l. The intra-assay coefficient of variation was 5.9%. For the C kit the cross-reactivity of the antiserum was less than 1% with all naturally occurring steroids, with the exception of 11-deoxycortisol (11.4%), prednisolone (76%) and prednisone (2.3%). The sensitivity of the assay was 5.5 nmols//l. Intra-assay coefficient of variation was 7.4%. To correct for the effects of varying fluid intake in the animals creatinine concentrations of urine samples were determined as described by Malherbe et al. [60]. The intra-assay coefficient of variation for creatinine was 3.1%.

### Assessment of the Possible Impact of Co-infection

During this experimental period sawdust was replaced with paper towels as bedding to facilitate collection of food remains (see below). For three days prior to injection the body mass (pre-treatment body mass) was assessed for each individual daily as described above. In addition, each individual received 60 g of fresh sweet potato daily. Any uneaten food was removed daily and dried to constant weight. Similarly, we dried 20 pieces of sweet potato of 60 g fresh weigh each to a constant weight to determine a quotient that allowed us to calculate the amount of fresh sweet potato eaten daily from the dried up food remains (wet weight = 17.8*dry weight). Food intake correlated significantly with body mass on any given day (R = 0.504, p<0.0001) and we thus corrected food intake for body mass. After this baseline period animals were injected subcutaneously with a dose of 1 mg kg^-1^ LPS (from *Escherichia coli* serotype 026:B6, Sigma Chemical) dissolved in sterile 0.9% saline or the equivalent volume of saline. This dose was chosen based on previous experimental LPS-challenges in the study species [57]. Body mass as well as food intake was assessed for the day of treatment (day 1) as well as the two following days (day 2 and 3). The time window was chosen based on preliminary experiments that employed data collection over five days pre- and post-injection as they suggested no significant changes in the measured variables beyond day 3 post-injection. Eight uninfected animals received a saline injection (control-S) while the remaining 12 uninfected males (control-LPS) and all infected males (infected-LPS) were treated with LPS. On completion of the experiment animals again were maintained on saw dust and received an *ad libitum* diet of sweet potato.

### Haematology

Animals were euthanized with an overdose of halothane within two weeks of completion of the experiment and body mass was recorded once again (final body mass). The body cavity was opened, a blood sample was obtained through exsanguination from the heart and stored in heparinised microtubes. The red blood cell count (RBC) and levels of haematocrit for red blood cells and the proportion of neutrophils, lymphocytes, monocytes, basophils and eosinophils as white blood cell differentials was assessed with an automated counter within 4 hours of collection at the Onderstepoort Veterinary Hospital. In addition, we calculated the ratio of neutrophils to lymphocytes (N:L) as a measure of stress [61]. Some of the blood samples were clotted and thus sample sizes were reduced for the control-LPS and the infected-LPS group (see Table 1). No blood samples were collected at the beginning of the study to avoid confounding effects on cortisol measures. We also recorded the mass of liver, kidney, heart, spleen and fat deposits to the nearest 0.01 g.

### Statistical Analyses

To assess the effect of treatment and cestode infection on overall body mass (initial vs. final), hormonal levels, body mass changes and food intake we employed linear mixed models (GLMMs). We included individual ID as random factor in all models to account for the repeated measurement of males. In addition, treatment (saline or LPS-injection) and parasite status (i.e. uninfected/infected) were included as fixed factors in all models. For the overall body mass changes we also included time (initial vs. final) of measurement. Similarly, for the hormones, time was included as factor (pre- vs. post-injection) while we included experimental day (pre, day 1, day 2 or day 3 post-injection) as factors for the models that had body mass loss and food intake, respectively, as dependent variables. All main effects and the interaction between treatment and time and between time and infection status were included in the model and post-hoc tests were conducted for significant interaction terms using the least-significant (LSD) difference. Values for both T and C had to be square-root transformed prior to analysis to satisfy the requirements of parametric data. Neither pre-treatment body mass nor food intake differed significantly between days (p≥0.27), hence, we used the means of the respective measures during the pre-challenge period for comparisons. Posthoc power analyses were carried out in order to evaluate the possibility that the null hypothesis (there was no difference between infected and uninfected males) was wrongly accepted (β) using the program GPower [62]. We assessed the effects of parasite infection and treatment on organ masses employing general linear models (GLMs) with treatment (saline or LPS-injection) and parasite status as independent variables. fat and spleen mass had to be log-transformed to satisfy the criteria for a parameteric test. These analyses were repeated including body mass as covariate to control for any confounding effects of body mass. Similarly, the effect of treatment and parasite infection on RBC, haematocrits and lymphocyte counts were analysed with GLMs. However, since transformations remained unsuccessful we fitted a model with a gamma-distribution and a log-link function for the analyses of neutrophils and N:L, while a Poisson-distribution and log-linear function were fitted for monocytes, eosinophils and basophils that had very low counts. In all these models treatment and parasite status were included as independent variables. Results are presented as means ± SE.
